# Supplementary material for: Patterns of Intron Gain and Loss in Fungi
Source: PLoS Biol. 2004 Nov 30;2(12):e422. doi: 10.1371/journal.pbio.0020422 (PMC532390; doi:10.1371/journal.pbio.0020422)
Supplement: Table S1 — Also available at http://genes.mit.edu/NielsenEtAl/. (4.3 MB ZIP). [file pbio.0020422.st001.zip › NielsenEtAl/html/352.html]

AN0351.1.NCU00742.1.MG00067.1.FG05023.1


```
 CLUSTAL W (1.82) Multiple Sequence Alignments - Introns Inserted


Sequence 1: MG00067.1	433 aa
Sequence 2: FG05023.1	417 aa
Sequence 3: AN0351.1	417 aa
Sequence 4: NCU00742.1	513 aa
Alignment Length: 551 aa
Number Identitical Residues: 171 aa
Alignment Score (without introns) 9282


MG00067.1 	--~---------------------------------------------------------
NCU00742.1	MY0ARYRSRILSSDAASVFHYLVQGQGPKLPLSFSSPPTHSALRRRFYVQSSAAISKPTS
FG05023.1 	--~---------------------------------------------------------
AN0351.1  	--~---------------------------------------------------------
          	                                                            

MG00067.1 	-------------------------------MASLGSYA---------------------
NCU00742.1	PFHQQLPRSPQIATRADVLEPASSLWSTRSSRASLAPPLNNTRHLPVRRYSQVAKMTVNN
FG05023.1 	-------------------------------MASLGAPS---------------------
AN0351.1  	-------------------------------MGSLGPYK---------------------
          	                                .**..                       

MG00067.1 	--KKHKVTIIGSGNW~GSTIAKIVAESTREHKDVFEEDVQMWVFEEKVTIPKDSPYYESE
NCU00742.1	FIKKHKVTVIGSGNW~GTTVAKLVAENCAEHPDTFEKDVQMWVYEENVTLPKDSKHFDAS
FG05023.1 	--KKHKVTIVGSGNW2GSTIAKIVAENTRAHKELFEEDVQMWVYEENVTIPKESKLYDEA
AN0351.1  	--QKHKVTVVGSGNW2GTAIAKIVAENTASNPAVFEKDVQMWVFEEKVEIPKSSKHYDPA
          	  :*****::***** *:::**:***.   :   **:******:**:* :**.*  ::  

MG00067.1 	E-----PQKLTEVINKHHENVKYLPGIKLPSNIIANPSLTDAVRDSSVLVFNLPHEFLGK
NCU00742.1	IGEQ--PQKLTHILNTYHENVKYLPGIPLPHNLVANPSLVDAVKDSSILIFNLPHEFIGN
FG05023.1 	NGDA--PQKLTEVINKHHENVKYLPGIPLPTNLIANPDIRDAVKDSSILVFNLPHQFIAN
AN0351.1  	SSLCQGPQNLTDIINHTHENIKYLPGITLPENLIANPSLVDAVQDSTILVFNLPHQFIIN
          	 .  ..**:**.::*  ***:****** ** *::***.: ***:**::*:*****:*: :

MG00067.1 	VCQQLNGHIVPFARGISCIKGVDVSGSGINLFCEVIGEKLGIYCGALSGANVASQIAAEE
NCU00742.1	ICRQIKDHIAPYARGISCIKGVSVTDDKIELICNYVSDALNIYCGALSGANIASEIANEQ
FG05023.1 	VCKQINGHILPFARGISCIKGVNVTDTDISLFSEWIGDGLGIYCGALSGANLAREIAEEK
AN0351.1  	ICEQIKGKIVPYARGISCIKGVDVNEEGVHLFSETIGKILGIYCGALSGANIANEVAQEK
          	:*.*::.:* *:**********.*.   : *:.: :.. *.**********:* ::* *:

MG00067.1 	GVSETTIAYDPPPIDSSRAATPRDRSPNYDSTSANKLPDLTVTSADSNGKDDRGRRTKAK
NCU00742.1	-WCETTIAYNTPLVDRRDENGRPVGNG--------------------ESND-----HTKQ
FG05023.1 	-WSETTIAYDPPALDNSRAPTPRSGSPN---PTMGELP-------EAHHKDVRGRTSKTK
AN0351.1  	-WSESSIGYDPPHFDS-KAPSPPNRSPS---ASTDNIL-------HFEHKDVSGQLSRVK
          	  .*::*.*:.* .*          . .   .: ..           . :*  .     :

MG00067.1 	LTPVPESYPPLDHGTLQILFDRPYFSVSMVSDVAGVSLSGALKNIVALAAGFVDGKGWGS
NCU00742.1	LTPLPLDYPPLDHDEWSLLFSRPYFTVSMVSDIVGVSLAGALKNIVAIACGYVEGHGWNM
FG05023.1 	LTAMPAEYPPLDQHCFRTLFHRPYFHVQMVSDVAGVSLSGALKNVVALAAGFVDGRGWGD
AN0351.1  	LQALPSEFPPIDHALLKSLFHRPYFHIGVVSDVAGVSLGGALKNVVAVAAGWVVGKGWGD
          	* .:* .:**:*:     ** **** : :***:.****.*****:**:*.*:* *:**. 

MG00067.1 	NVQSAVIRVGLAEMLKFAREFFGESVDPFTILLESAGVADVITSCISGRNFRCASMAVKR
NCU00742.1	TAKTAVMRRGMQEIIRFANEFFPGQIEPRTFWEESAGWGDMIVSCTSARNWRCSKLAVER
FG05023.1 	NAKAAIMRVGLMEMVKFGKEFFGETVHTATFTESSAGVADLITSCSGGRNFRCAKMAVQK
AN0351.1  	NAKAAIMRVGLLEMVKFGEQFFGATINTRTFTEESAGVADLITSCSGGRNFRCAKLSIER
          	..::*::* *: *:::*..:**   :.. *:  .*** .*:*.** ..**:**:.:::::

MG00067.1 	GVSVAEIEEKELNGQKLQGTSTAKEVNSLLKARGREGDYPLFTTVNE1ILEGKARVDDLP
NCU00742.1	NLPIEEVEKTELNGQKLQGISTTREVSSFLRAKGAEERYPVFMAVER~IIDQEIKVEEIP
FG05023.1 	GISVQEVEKQELNGQKIQGTTTAEEVNSFLKARGLESEYPLFTAVNA~ILNGEAQVDDIP
AN0351.1  	NQPIEKIEETELNGQKLQGTLTAVEVNSFLKKQGLEEEFPLFTAVYR1VLQGTMSVDEIP
          	. .: ::*: ******:**  *: **.*:*: :* *  :*:* :*   :::    *:::*

MG00067.1 	KLVIRQKHTIEKSG
NCU00742.1	KLFK----------
FG05023.1 	TLIQDS--------
AN0351.1  	SFIER---------
          	.:.
```
